# Safety Evaluation of Natural Drugs in Chronic Skeletal Disorders: A Literature Review of Clinical Trials in the Past 20 years

**DOI:** 10.3389/fphar.2021.801287

**Published:** 2022-01-13

**Authors:** Dongyang Zhou, Hao Zhang, Xu Xue, Yali Tao, Sicheng Wang, Xiaoxiang Ren, Jiacan Su

**Affiliations:** ^1^ Institute of Translational Medicine, Shanghai University, Shanghai, China; ^2^ Institute of Advanced Interdisciplinary Materials Science, Shanghai University, Shanghai, China; ^3^ College of Medicine, Shanghai University, Shanghai, China; ^4^ College of Environmental and Chemical Engineering, Shanghai University, Shanghai, China

**Keywords:** natural drugs, chronic skeletal disorders, ageing, clinical trial, safety evaluation

## Abstract

Chronic skeletal disorders (CSDs), including degenerative diseases such as osteoporosis (OP) and autoimmune disorders, have become a leading cause of disability in an ageing society, with natural drugs being indispensable therapeutic options. The clinical safety evaluation (CSE) of natural drugs in CSDs has been given priority and has been intensively studied. To provide fundamental evidence for the clinical application of natural drugs in the elderly population, clinical studies of natural drugs in CSDs included in this review were selected from CNKI, Web of Science, PubMed, Science Direct and Google Scholar since 2001. Seventeen randomized controlled trials (RCTs) met our inclusion criteria: four articles were on OP, seven on osteoarthritis (OA), four on rheumatoid arthritis (RA) and two on gout. Common natural drugs used for the treatment of OP include *Epimedium brevicornu Maxim [*Berberidaceae*]*, *Dipsacus asper Wall ex DC [*Caprifoliaceae*]* root, and *Phalaenopsis cornu-cervi (Breda) Blume & Rchb. f[* Orchidaceae*]*, which have been linked to several mild adverse reactions, such as skin rash, gastric dysfunction, abnormal urine, constipation and irritability. The safety of *Hedera helix L [*Araliaceae*]* extract, *Boswellia serrata Roxb [*Burseraceae*]* extract and extract from perna canaliculus was evaluated in OA and upper abdominal pain, and unstable movements were obsrerved as major side effects. Adverse events, including pneumonia, vomiting, diarrhoea and upper respiratory tract infection, were reported when RA was treated with *Tripterygium wilfordii, Hook. F [*Celastraceae*]*[TwHF] polyglycosides and quercetin (*Capsella bursa-pastoris (L.) Medik [*Brassicaceae*]*). The present review aimed to summarize the CSE results of natural drugs in CSDs and could provide evidence-based information for clinicians.

## Introduction

The skeletal system has pivotal physiological functions, such as maintaining body shape (Gordon and Gordon, 2020), participating in limb movement, protecting important organs and detoxification (Londzin et al., 2020). The difficulty of healing with ageing is a main feature of CSDs, which has become a growing worldwide public health concern (Peng et al., 2021; Jaschke et al., 2021). The incidence of these chronic diseases is also impacted by the improvement in living standards and lack of exercise (Linbi et al., 2019). Age-related bone loss and joint degeneration are common causes of CSDs, such as OP and arthritis (Sebastian et al., 2020; Ju et al., 2021). Chemical drugs play a predominant role in the clinical treatment of these CSDs due to their rapid effects (Rodan and Martin, 2000; Gordon and Gordon, 2020). However, it has been observed that chemical drugs have many adverse effects (Lien et al., 2021). For example, Gupta, et al. (Gupta, et al., 2019) reported bisphosphonates are used to treat OP, resulting in adverse reactions such as o oily skin, fluid retention, nausea, long-term toxicity, and even prostate cancer in males; Christian et al. (Christian et al., 2021) reported NSAIDs would lead to cardiovascular, renal and gastrointestinal events in the elderly. Low metabolism and healing rates amplify the side effects of chemical drugs, which could subsrequently engender serious secondary injury, especially in elderly patients (Molina et al., 2014; Colón et al., 2018). The search for and study of drugs for CSDs with fewer side effects have been a lifelong focus of many researchers.

Natural drugs are those obtained from animals, plants and minerals that have certain proven pharmacological activity by modern pharmaceutical systems (Ma et al., 2019; Chang et al., 2020). Natural drugs are increasingly favoured by researchers and clinicians for their lower side effects and adverse reactions (Zeng and Jiang, 2010). Some natural drugs have been used to treat chronic skeletal disease since ancient times (Chen et al., 2016; Li et al., 2021). Natural drugs can be classified according to the different CSDs they treat: 1) OP, for instance, lcariin (Zhai et al., 2013; Xu et al., 2016; Wang Z. et al., 2017), *D. asper Wall ex DC* root (Zhan et al., 2009; Su et al., 2018), resveratrol (Wang X. et al., 2017; Feng et al., 2017; Yang et al., 2019), quercetin (*C. bursa-pastoris (L.) Medik*) (Zhen et al., 2018), ginsenoside (Yang et al., 2020) and genistein (Chen et al., 2018); and 2) OA, for instance, curcumin (Sun et al., 2017), capsaicin (Persson e t al., 2018), berberine (Wong et al., 2019), and *TwHF* polyglycosides (Lindler et al., 2020). Recent developments in CSDs research have heightened the demand for natural drugs.

To maximize the benefits and minimize the toxicity of natural drugs, safety evaluation has become an indispensable component of natural drug research (Si et al., 2017; Silva and Pogacnik, 2020). This article not only provides the CSEs of natural drugs in treating CSDs but also provides a comprehensive reference for natural drug research in skeletal disease.

### Natural Drugs for Osteoporosis

Osteoporosis (OP) is a systemic bone disease characterized by decreased bone density and bone mass due to different causes, and this disruption of bone microstructure increases bone fragility (Jarvinen and Kannus, 2019; Wang et al., 2020), such as that seen in postmenopausal OP, senile OP and idiopathic OP (Ann et al., 2019; Jarvinen and Kannus, 2019). As ageing is aggravated in developed countries and in China, the incidence of OP is increasing sharply and has become an assignable health problem (Cui et al., 2019; Fan and Xia, 2019).

Lu Min et al. (Lu et al., 2013) designed an RCTs in patients from different regions to evaluate the clinical safety of the total flavones in *E. brevicornu Maxim.* capsules in treating primary OP. A random sample of 480 patients with primary OP was recruited and divided into two groups. The experimental group with 360 cases was prescribed total progesterone 0.7 g three times per day, while the control group with 120 cases was prescribed Gusongbao capsule 1 g three times per day. After 2 years of follow-up, the results revealed that the total efficacy of the experimental group and control group was 90.83 and 75.00%, respectively. The safety evaluation revealed that 1) no significant abnormalities occurred in noticeable parameters such as blood pressure, heart rate and body temperature after medication; 2) during the trial, no significant abnormalities were found in routine blood, urine and stool tests, including blood ALT, BUN, and Cr tests and electrocardiogram; and 3) twenty-four adverse events happened in the group receiving the total flavones from *E. brevicornu Maxim* capsules with an incidence of 6.67%. These adverse events were rash, dizziness, constipation, diarrhoea, palpitations, tinnitus, abdominal pain, sore stomatitis, ulcerative stomatitis, gastric dysfunction, pharyngitis, reduced sweating, and abnormal urine. Moreover, the control group had six adverse events with an incidence of 5.00%; these events included constipation, stomatitis, and ulcerative stomatitis.

In a multicentre, randomized, double-blind, parallel controlled clinical trial, Zhan Hongsheng et al. (Zhan et al., 2009) divided 600 volunteers into *D. asper Wall ex DC* capsule group (total saponin extract of *D. asper Wall ex DC* 0.28 g/tablet) and two other control groups (experimental group) at a ratio of 3:1:1. The total effective rate of experimental group was 86.82%. The safety analysis results after 6 months indicated that 12 adverse events occurred within the trial group, with an incidence rate of only 3.33%; among them, there were 6 cases of abdominal discomfort with an incidence rate of 1.67%; 3 cases of constipation with an incidence rate of 0.83%; and 1 case of dysphoria, 1 case of swollen gums and 1 case of elevated blood ALT levels, each with an incidence rate of 0.28%. All of the above symptoms were mild adverse reactions.

Wang Hong et al. (Wang et al., 2004) reported clinical efficacy obserervations of *P. cornu-cervi* (The young horns of a male deer are not ossified and densely hairy) capsule in treating primary OP. Sixty OP patients were divided into two groups based on different treatments: *P. cornu-cervi* capsule (A group) or Gushukang capsule (B group) for 12 months. The total effective rate of group A was 82.14%, while the total effective rate of group B was 67.86%. Drug safety analysis showed that there was no heart, liver, or renal function damage, and electrocardiography of four cases that experienced myocardial strain returned to normal after *P. cornu-cervi* capsule treatment. Interestingly, two patients with abnormal routine urinary tests became normal after *P. cornu-cervi* capsule treatment. Nevertheless, one patient had abnormal counts of leukocytes in the routine urinary test but returned to normal after norfloxacin treatment. No adverse effects of this drug on routine blood, stool or electrolyte tests were found during treatment. Interestingly, the WBC count of one case in routine blood tests rose from 2,700 to 4,600. Except for one case in the treatment group who withdrew from the experiment due to severe stomach pain, the majority of the volunteers tolerated the treatment very well.

Unfer et al. (Unfer et al., 2004) conducted a long-term randomized, double-blind, placebo-controlled trial to estimate the clinical safety of Phytoestrogens from *Glycine* max *(L.) Merr [*Fabaceae*]* in the treatment of postmenopausal OP. Three hundred seventy-six healthy postmenopausal women with intact uteri participated. One hundred seventy-nine patients in Group A received soy isoflavones (150 mg/d), and 197 patients in Group B received placebo for 5 years. However, There was no report on the effect of treatment, the safety results showed that among 298 female patients who completed 5 years of treatment, no malignant pathological changes were detected at the time of biopsy. Seventy percent of women receiving Phytoestrogens from *G.* max had atrophic or unevaluable changes in the endometrium, compared to 81% in the placebo group. The incidence of endometrial hyperplasia was higher in Group A than in Group B (3.37 vs 0%). Long-term administration of Phytoestrogens from *G.* max (up to 5 years) is associated with an increased incidence of endometrial hyperplasia. The above results suggest that the long-term impact and safety of phytoestrogens on the endometrium of postmenopausal women with OP should be considered.

From these clinical trials, it was found that the probability of adverse events of several natural drugs (*E. brevicornu Maxim.*), Himalayan teamel root; *P. cornu-cervi.* and Phytoestrogens from *G.* max was lower than that of the control group, indicating that the safety of those drugs was likely higher. Taken together, these results suggest that natural drugs could become a potential choice in the treatment of OP.

### Natural Medications for Arthritis

Arthritis also belongs to the CSDs that affect load-bearing joints (knee, hip, foot and spine), joint synovium, periarticular bone and adjacent supporting connective tissue. The incidence of arthritis has become increasingly difficult to ignore in the elderly population and is expected to be a leading cause of worldwide population disability by 2030 (Glyn-Jones et al., 2015). The safety assessment of natural medicines for the treatment of OA, RA and gout is arranged in here to describe.

### Osteoarthritis

Primary Osteoarthritis (OA) is widespread in middle-aged and elderly individuals, affects load-bearing joints and is characterized by articular cartilage degeneration, subchondral bone sclerosis, osteophyte formation and bone marrow oedema (Hunter and Bierma-Zeinstra, 2019). The clinical symptoms are mainly joint stiffness, joint pain, limited range of joint movement and joint deformity (Hunter and Bierma-Zeinstra, 2019; Glyn-Jones et al., 2015). The pathogenesis of OA is so unclear that the main treatment methods are simply aimed at relieving joint pain and at postponing joint replacement. Acesodyne, such as some nonsteroidal anti-inflammatory drugs, have side effects in the gastrointestinal, cardiovascular, and renal systems (Glyn-Jones et al., 2015). Clinical research on natural drugs has brought novel and safe therapeutic targets for the treatment of OA.

Pagosid is a pure natural plant preparation extracted from the massive roots of a plant called “devil’s claw” (*Harpagophytum procumbens (Burch.) DC. ex Meisn* [Pedaliaceae]) growing in the Kalahari desert of Southwest Africa and the grassland of Namibia (Qiyun, 2001). It is mainly used for the treatment of rheumatic diseases and all kinds of pain. Liao Qiande et al. (Liao, et al., 2011) designed a clinical trial study to obsrerve the safety of pagosid and selected 268 patients with primary knee OA. Oral pagosid (3 × 820 mg/d, Swiss SmithKline pharmaceutical factory) treatment was administered for 8 weeks to observe knee tenderness, activity pain, joint swelling and daily activity at 4 and 8 weeks, as well as 4 weeks after drug withdrawal. The results indicated that the total efficacy was 65.8% at week 4, 80.8% at week 8, and 72.4% at 4 weeks after drug withdrawal. The incidence of adverse reactions, including digestive system, central nervous system and circulatory system events, was only 6.4% after 8 weeks of treatment. Specifically, there were 4 cases of dizziness, 6 cases of abdominal distension, 5 cases of nausea and vomiting, 1 case of giddiness, and 3 cases of abdominal discomfort. This research showed low adverse reactions and side effects of pagosid.

Morteza Dehghan et al. (Dehghan et al., 2020) published a comparison of the therapeutic effects of *H. helix L.* extract gel and diclofenac gel on knee OA. One hundred fifty (150) patients with primary OA were randomly assigned into three groups, namely, the 1% *H. helix L.* extract gel treatment group, 1% diclofenac gel treatment group, and placebo treatment group. Drugs were applied orally times a day, for 3–5 min each time, during a trial period of 6 weeks. All patients were allowed to take celecoxib capsules daily. The efficacy results revealed that the groups treated with 1% *H. helix L.* extract gel or 1% diclofenac gel had a significantly higher easement of pain than the placebo group. The safety analysis showed that the 1% *H. helix L* extract gel-treated group did not show any allergic reactions or adverse reactions compared to those of the 1% diclofenac gel-treated group. However, the 1% diclofenac gel-treated group exhibited a significant decrease in body function.

Marzieh Alazadeh et al. (Alazadeh et al., 2020) conducted an RCTs to obsrerve the effect of *Foeniculum vulgare Mill [*Apiaceae*]* (commonly known as Fennel) seed extract capsules in relieving knee OA pain. Sixty-six 66) patients were randomized to the sweet fennel seed extract capsule group and the placebo group. Treatment consisted of oral intervention of fennel extracts (4 capsules containing 800 mg of dry fennel extract from 28 g of fennel seeds) or placebo twice a day for 2 weeks. Oral fennel can reduce Western Ontario and McMaster Universities Osteoarthritis Index (WOMAC) pain by about 36%, Visual Analog Scale (VAS) pain by about 32%, and the placebo group’s pain reduction rates were 15 and 13%, respectively. These results indicate that fennel is effective in controlling the symptoms of knee OA. The safety analysis found that no serious side effects occurred, but a transient effect on the breast appeared in the drug intervention group. Heartburn was another side effect reported by three patients in the placebo group and two patients in the fennel group; these subjects were subsequently excluded from the study.

B. Grube et al. (Grube et al., 2007) reported the therapeutic effect of a comfrey root (*Symphytum officinale L [*Boraginaceae*]*)extract ointment on OA pain. This trial adopted a double-blind, double-centre, placebo-controlled randomized trial that recruited 220 patients with knee OA at an average age of 57.9 years. Patients were randomized into two groups with Kytta Salbe containing purple grassroots liquid extract or the control group. Both of the groups took drugs orally three times daily, 2 g each time for 3 weeks. The treatment effect was evaluated by the total score of VAS and the total scores of the WOMAC. The results suggest that a comfrey root extract ointment can reduce pain and improve mobility in the treatment of knee osteoarthritis. The safety evaluation depicted that 22 adverse reactions (10.0%) were reported, including 7 cases in the trial group (6.4%) and 15 cases in the control group (13.6%). All of the adverse reactions were mild symptoms, and no significant difference was found between the two groups. The authors considered that there were no obvious adverse reactions to the drug. Patients and doctors were questioned four times during the trial to comprehensively assess drug tolerance. Responses were classified as ‘very good’, ‘good’, ‘moderate’ or ‘bad’. Among them, ‘very good’ was the response for 73.6% (doctors and patient) in the trial group.

Vilai Kuptniratsaikul, M.D. et al. (Kuptniratsaikul et al., 2009) reported the treatment effect and safety analysis of Curcuma domestica (*Curcuma longa L [*Zingiberaceae*]*) extract on the alteration of pain and joint function in knee OA patients. One hundred seven patients (107) with OA were randomized into 55 controls who took ibuprofen 800 mg a day, and 52 patients in the *C. domestica L.* extract group who took curcumin 2 g per day for 6 weeks. The time of walking 100 m, going up and down stairs was used to evaluate the improvement of horizontal walking pain, stair pain and knee function. The therapeutic effect of *C. domestica L.* extracts is almost consistent with that of ibuprofen for the treatment of keen OA. Then, the safety analysis results are shown in Table 1. Sixteen adverse reactions (33.3%) occurred in the curcumin group, while only three occurred in the ibuprofen group.

**TABLE 1:** Adverse reactions to curcuma extracts in knee OA and ibuprofen patients.

	*Curcuma domestica* extract group (*n* = 48)	Ibuprofen group (*n* = 52)	*p* -value
Total No. of patients with an AE	16	23	0.35
Alimentary system	15	21	
Dizziness	5	2	
Dry mouth	0	2	
Rash	0	1	
Fatigue	0	1	

As we can see from Table 1, the adverse reactions included indigestion, dizziness, nausea and vomiting, and loose stool. No significant changes were found in the blood tests at 0 weeks or 6 weeks. The tolerance of the ibuprofen group was better than that of the *C. domestica L.* extract group (90.1 vs 82.8%, *p* = 0.001), which may be because of the odour of the *C. domestica L.* extract.

N. Kimmatkar et al. (Kimmatkar et al., 2003) reported the efficacy and tolerance of *B. serrata Roxb* extract (BSRE) in knee OA using RCTs. Thirty (30) knee OA patients were divided into the BSRE and placebo groups. All patients took capsules of the same appearance and weight three times per day, one capsule each time for 8 weeks, and the BSRE group capsules contained 333 mg of BSRE. The clinical efficacy suggest that compared with the placebo group, the reduction of pain and swelling severity and the improvement of functional loss in the active drug group were clinically and statistically significant (*p* < 0.001). Safety analysis revealed that adverse reactions included one instability of gait and one upper abdominal pain with nausea, and neither of them quit the trial due to adverse reactions. The overall compliance of patients to BSRE was acceptable.

Simon Stebbings et al. (Stebbings et al., 2017) reported the efficacy and safety of extract from the BioLex®-Green Lipped Mussel (BioLex®-GLM) (*perna canaliculus*) for treating pain in the hip and knee OA. This trial selected 80 patients with moderate to severe hip or knee OA pain and randomized them to two groups treated with 600 mg of BioLex®-GLM per day or placebo for 12 weeks. This biolex®-GLM extract treatment results only showed less acetaminophen than the placebo group, because biolex ®- GLM extract can reduce pain in patients with moderate and severe arthritis. In addition, the treatment group also significantly reduced the degree of stiffness. Safety analysis showed that three adverse events occurred in the placebo group: 1) systemic pain and flu-like symptoms 7 weeks after treatment with placebo, 2) a bilateral pulmonary embolism after 24 days of placebo administration, and 3) extensive generalized pruritus rash after 62 days of placebo, with the rash lasting for 3 weeks. Only one adverse event occurred in the Biolex®-GLM treatment group but required hospitalization because of abdominal pain that occurred 8 days after treatment started. The author found that the patient had a chronic history of stomach disease, which means that the adverse event may not have been caused by Biolex®-GLM. All of the above volunteers withdrew from the study after the adverse reactions occurred.

All the results of clinical trials indicated that natural drugs have good safety profiles and obvious treatment effects and are expected to become alternative drugs for the treatment of OA.

### Rheumatoid Arthritis

Rheumatoid arthritis (RA) is an autoimmune disease in which the immune system is activated abnormally and attacks healthy joints (Brennan et al., 2019; Edmonds et al., 2019; Mutru et al., 2019) and affects over 21 million people worldwide (Aletaha and Smolen, 2018). Hyperplasia of synovial tissue invades and damages articular cartilage and is the main characteristic of RA (Rosa et al., 2020). Symptoms of RA include weakness, fever, fatigue, weight loss and myalgia (Aletaha and Smolen, 2018). Females are more vulnerable to RA than males (Alpizar-Rodriguez et al., 2017).

(Goldbach-Mansky et al., 2009; Raphaela Goldbach-Mansky et al., 2021) compared the effect and safety of TwHf extracts and sulfasalazine on RA by randomized controlled trials. One hundred twenty-one 121) patients with active RA were selected, and more than six patients had joint pain and swelling. Participants took 3 × 60 mg/d TwHf in the TwHf group or sulfasalazine 1 g twice per day in the control group. After 2 weeks of treatment, a significantly greater improvement was found in the TwHF group compared with the sulfasalazine group at baseline, and this improvement continued throughout the assessment of health, ESR and CRP levels. From 8 weeks of treatment, the improvement in the number of swollen and tender joints in TwHF group was statistically significantly greater than that in sulfasalazine group. Adverse events are illustrated in Table 2; approximately 60% of all patients and volunteers had gastrointestinal symptoms. Fifteen serious adverse events were reported in 10 patients, three of whom received TwHf treatment and seven of whom received sulfapyridine. Seventeen patients in the sulfadiazine group dropped out due to adverse events, while only eight patients in the TwHf group dropped out. Adverse events that led to seceding from the study included six gastrointestinal events, one thrombus reduction, one severe adverse event and one femur fracture. It is obvious that patients in the sulfasalazine group had moderate to severe adverse events compared to those of the TwHf group.

**TABLE 2:** Comparison of adverse reaction events between TwHf and sulfasalazine in the treatment of RA (Goldbach-Mansky et al., 2009), Copyright 2009, ACP.

Adverse events	TwHf all patients (n = 60)	Sulfasalazine group all patients (n = 61)	*p* Value
Total events, n (%)			
Any event	53 (88.3)	55 (90.2)	0.78
Related to study drug	34 (56.7)	37 (60.7)	0.71
Serious adverse events‡	3 (5)	7 (11.5)	0.32
Most frequent adverse events, n (%)			
Nausea	13 (22)	21 (34)	0.157
Vomiting	9 (15)	9 (15)	1.00
Diarrhoea	15 (25)	11 (18)	0.38
Constipation	5 (8)	8 (13)	0.56
Dyspepsia	13 (22)	15 (8)	0.044
Abdominal distention	6 (10)	2 (3)	0.163
Abdominal pain	11 (18)	6 (10)	0.20
Infectious adverse events			
Upper respiratory tract infection	11 (18)	6 (10)	0.20
Influenza	2 (3)	4 (7)	0.68
	1 (2)	0 (0)	0.50
Urinary tract infection	2 (3)	6 (10)	0.27
Pneumonia Other infections	2 (3)	7 (11)	0.163

Qian-wen Lv et al. (Lv et al., 2015) conducted a clinical trial to compare the safety between methotrexate (MTX) and TwHf in treating active RA (TRIFRA). This was a multicentre, open label and randomized controlled trial. Two hundred twenty-seven 227) active RA patients were recruited and were assigned randomly and equally to three groups to receive MTX 12.5 mg once weekly, TwHf 20 mg three times a day or a combination of both treatments in 2 years. After treatment, the erythrocyte sedimentation rate (ESR) in TwHF group and combination group decreased significantly at week 12, while ESR in MTX group did not decrease significantly until week 24. In the fourth week, the TwHF group had a greater improvement in ESR changes than the MTX group (*p* = 0.04). Then, the safety evaluation results are summarized in Table 3. The analysis revealed that 52.7% of the patients had adverse events in all groups. The incidences of adverse events were 46.4, 62.3 and 49.3% in the TwHf group, MTX group and TwHf + MTX group, respectively. Gastrointestinal tract adverse effects, as the most common adverse reactions, appeared in 29.0, 43.5 and 34.8% of the TwHf group, MTX group and TwHf + MTX group (*p* = 0.202), respectively. One patient in the TwHf group, three patients in the MTX group and three patients in the TwHf + MTX group dropped out due to severe adverse events, but there was no statistically significant difference in the quit rate among the treatment groups (*p* = 0.554). Patients in the TwHf group dropped out due to elevated blood alanine transaminase (ALT 92 U/L). In the combination group, tuberculous pleurisy, elevated blood ALT (ALT 89 U/L) and a gastrointestinal event resulted in withdrawal. In the 24 weeks of the trial, 15 (8.8%) female patients (n = 170, including 69 premenopausal women) had menstrual irregularities, including seven patients in the TwHf group, three patients in the MTX group and five patients in the combined group (TwHf group vs MTX group, *p* = 0.216).

**TABLE 3:** Clinical adverse reaction events of *Tripterygium wilfordii* Hook F Multiglycosides (TwHf) and methotrexate (MTX) in the treatment of motor-active rheumatoid arthritis (Lv et al., 2015), Copyright 2014, Ltd (and EULAR).

Adverse events	MTX (n = 69)	TwHf (n = 69)	MTX + TwHf (n = 69)	Total events (n = 207)
All	43 (62.3)	32 (46.4)	34 (49.3)	109 (52.7)
Gastrointestinal, n (%)	30 (43.5)	20 (29.0)	24 (34.8)	74 (35.7)
Nausea	7	6	10	23
Vomiting	1	2	1	4
Loss of appetite	10	1	6	17
Diarrhoea	3	2	1	6
Abdominal distention	2	2	3	7
Abdominal discomfort	11	7	8	26
ALT elevation	11	4	6	21
Infection, n (%)	10 (14.5)	3 (4.3)	7 (10.1)	20 (9.7)
Upper respiratory tract infection	3	1	0	4
Skin and mucous event, n (%)	14 (20.3)	7 (10.1)	14 (20.3)	35 (16.9)
Irregular menstruation, n (%)*	3 (5.1)	7 (12.5)	5 (9.1)	15 (8.8)
Skin and mucous event, n (%)	14 (20.3)	7 (10.1)	14 (20.3)	35 (16.9)
Other adverse events, n (%)	16 (23.2)	5 (7.2)	13 (18.8)	34 (16.4)
Fatigue	5	2	1	8

According to the TRIFRA study, using TwHf alone is not inferior to the MTX + TwHf combination treatment, but combination treatment is better than MTX monotherapy in patients with active RA. These efficacy results confirmed that TwHf combined with methotrexate is a safer and effective treatment for active RA.

Shahin et al. (Khadem Azarian et al., 2019) reported the efficacy and safety of guluronic acid (G2013) from gulose (*Tinospora cordifolia (Willd.) Hook. f. and Thomson [*Menispermaceae*]*) through a randomized, double-blind clinical trial that recruited 52 R A patients. Twenty-six 26) patients were in the G2013 group, and the rest of the patients were in the control group. Both treatments significantly improved most primary efficacy endpoints within 12 weeks of G2013 and conventional drug treatment, and the difference between baseline and endpoint measurements of all efficacy endpoints of G2013 was significantly greater than that of conventional treatment subjects, which implies that the therapeutic effect of G2013 is significantly better than that of the traditional drug group. The outcome demonstrated a good tolerance of G2013 treatment. The adverse event incidence of the control group was higher (76.9%) than that of the G2013 group (15.3%). None of the patients receiving G2013 dropped out due to adverse events, while two patients quit in the control group. In addition, most patients reported a significant change in mood after G2013 treatment. There was no statistically meaningful dissimilarity in the mean haematologic and biochemical values at 0, 4 and 12 weeks after trial initiation, indicating the good safety profile of G2013.

Fatemeh et al. (Javadi et al., 2017) reported that quercetin (*C. bursa-pastoris (L.) Medik*) could change the clinical symptoms and inflammatory factors of females with RA via RCTs. Fifty women with RA were recruited and treated for 8 weeks with 500 mg/d quercetin or placebo. During the study period, 10 patients quit the trial due to the need to increase the drug volume or switch to other drugs. Eventually, 20 people in each group completed the trial. The evaluation of therapeutic effect showed that the clinical symptoms, disease activity, HS-TNFa and health assessment questionnaire of patients with rheumatoid arthritis were significantly improved after supplementing 500 mg quercetin every day for 8 weeks. And no side effects were reported except for one stomach ache from a patient in the placebo group who was excluded from the study.

### Gout

Gout, a painful inflammatory arthritis, is caused by monosodium urate crystals being deposited in synovial fluid and other body tissues (Martinon et al., 2006). Hyperuricaemia has been identified as the primary cause of gout (Zimmet et al., 2019; Richette and Bardin, 2021). In Western countries, the prevalence of gout has increased over the past few decades, affecting approximately 1–2% of adult males (Dehlin et al., 2020). The main method for the treatment of gout and hyperuricaemia is to reduce serum uric acid levels (Solomon et al., 2018). Allopurinol (Crisp, 2021; Drugs, 2021) and febuxostat inhibitors are commonly used drugs to reduce the level of circulating urate. However, the side effects and adverse events of these drugs often reduce patients’ willingness to receive drug therapy (Crisp, 2021), so it is worth paying attention to the safety of natural drugs in gout treatment.

Colchicine is a botanical alkaloid originally extracted from the seeds and bulbs of *Colchicum autumnale L [*Colchicaceae*]* (McKenzie et al., 2021). The therapeutic effect of high-dose and low-dose oral colchicine on early acute gout attacks was reported by Robert A et al. (Terkeltaub et al., 2010) in a multicentre RCTs. One hundred eighty-four 184) patients were recruited. Therapeutic effects were reported in 28 patients (37.8%), 17 patients (32.7%) and 9 (15.5%) patients in the low-dose group, the high-dose group and the placebo group, respectively. Twenty-three patients (31.1%) in the high-dose group, 18 patients (34.6%) in the low-dose group and 29 patients (39.7%) in the placebo group took rescue drugs within the first 24 h after the trial began. The results show that the effect of low dose is not as good as that of high dose. Adverse events in the low-dose group were similar to those in the placebo group. High doses of colchicine were related to diarrhoea, vomiting and other AEs compared with low doses of colchicine or placebo. After using a high dose of colchicine, 40 patients (76.9%) developed diarrhoea, 10 patients (19.2%) developed severe diarrhoea, and nine patients (17.3%) developed vomiting. Meanwhile, only 23.0% of patients treated with low doses of colchicine developed diarrhoea.

Kubomura, D et al. (Kubomura et al., 2016) conducted 4-weeks RCTs to investigate the efficacy and safety of tuna (belongs to the *Thunnini* tribe and is a subgroup of the Scombridae family) extracts containing imidazole compounds in anti-hyperuricaemia treatment. Forty-eight males without gout but with slightly high levels of serum uric acid were randomized into the low-dose (supplement dose of 238.6 mg/d) and high-dose (supplement dose of 477.1 mg/d) tuna extract groups or the placebo group. In the process of treatment, the uric acid levels in the trial supplement (low-dose and high-dose tuna extract) group decreased significantly at weeks two and four compared with the placebo group. In addition, in the second week after the intervention, the uric acid level of the high-dose tuna extract test supplement group was significantly lower than other groups. However, the safety results revealed 44 adverse events, such as cold symptoms, stomach pain and headaches, in all groups. Since those adverse events were generally mild, none of them were inferred to be due to the study treatment. Furthermore, no physical or cardiovascular symptoms or haematologic abnormalities were found during the intervention and follow-up periods.

## Discussion and Outlook

Skeletal diseases have become the leading cause of disability in China, and the high quality of life demanded by elderly patients promotes the innovation of drugs for treating CSDs (Zhou M, et al., 2019). Chemical drugs have explicit pharmacological mechanisms and effects, and the side effects on other important systems or organs should not be ignored. The safety of drugs in CSDs therapy is the major concern because those diseases are not normally lethal. Severe adverse effects that may influence the circulatory system or central nervous system restrict the application of chemical drugs. Natural drugs are relatively safer than artificially synthesized drugs because of their natural sources but still need to be verified by the clinical results of evidence-based medicine.

In this review, we collected high evidence-level clinical results of natural drug application in CSDs, especially in OP and OA. The safety evaluation results of natural drugs in human clinical trials were summarized. This manuscript collected a total of 17 clinical trials on skeletal diseases using the RCTs method. Among them, there were four articles on the treatment of OP, seven articles on the treatment of OA, four articles on the treatment of RA and two articles on gout. This evidence supports the conclusion that natural drugs have better tolerance and fewer side effects in CSDs.

On the premise of ensuring the effectiveness of natural drugs, adverse events can almost be ignored in the report of clinical trials for the treatment of OP. For instance, 360 cases used total flavones of *E. brevicornu Maxim* capsule in a 2-years follow-up, and only 6.67% had adverse reactions, including rash, dizziness, constipation, and diarrhoea. In a clinical trial study of total saponin extract of *D. asper Wall ex DC* roots, it was found that only 12 events of 300 cases in the experimental group (total saponin extract 0.28 g/tablet) had adverse reaction events (abdominal discomfort and constipation) at the 6-month follow-up. After 12 months of the treatment with *P. cornu-cervi* for OP (30 cases), only one case had abnormal urine routine leukocyte count and returned to normal after norfloxacin treatment and one case of stomach pain were reported. We screened three natural drugs with the lowest incidence of adverse events. These natural drugs had enough safety for long-term administration. Among them, *P. cornu-cervi* had the best safety and has potential utilization value in the treatment of OP. On the other hand, the clinical reports discribing the treatment of natural drugs for osteoporosis were already 10 years ago. The clinical trials in recent years have not paid enough attention to the therapeutic effect of natural drugs, which hinders the development of natural drugs. In the future, more efforts should be made to the research on the use of natural drugs in the treatment of osteoporosis.

There are many studies on natural drugs for the treatment of OA. Here, we selected three natural drugs with high safety profiles, according to the number of adverse events from the seven natural drugs mentioned in this paper. After treatment with 1% *H. helix L.* extract for 6 weeks, 75 patients of all 1% *H. helix L* group did not report any adverse events. After 8 weeks of application of *B. serrata* extract (contained 333 mg/d of BSRE), there was only one case of instability of gait and one case of upper abdominal pain. Among 80 patients with OA treated with 600 mg of BioLex®-GLM per day or placebo for 12 weeks, only one case of abdominal pain occurred within 12 weeks due to a long-term history of stomach disease in that patient. In conclusion, the above three natural drugs for the treatment of OA have excellent safety and could be potential drugs for OA. By comparing these natural drugs, we found that few reports on adverse reaction events of these three natural drugs. Therefore, we have a hypothesis whether these drugs can still increase the dose (within a reasonable dose) to amplify the therapeutic effect. In addition, some natural drugs anti-inflammatory and pain-relieving effects can also be used for the clinical treatment of arthritis, because they are very corresponding to the symptoms of OA.

According to the statistics of clinical trials for the therapeutic effect of natural drugs in RA, the adverse event rate of *TwHF* polyglycosides (60 mg/d) accounted for 46% in 2 years. The incidence of adverse events in the G2013 group was 15.3% within 12 weeks, while there were no adverse events in the quercetin group for 8 weeks. Therefore, quercetin (*C. bursa-pastoris (L.) Medik*) is attractive for the treatment of RA because of the lack of adverse events. Many natural drugs had to stop clinical trials in view of serious adverse reactions, even they had good therapeutic effects. Therefore, there is a reliable inference that it may be difficult to find natural drugs with good therapeutic effect and low adverse side effects due to the complex pathological symptoms of RA.

According to the literature, there are few clinical trials of natural drugs for the treatment of gout, and only two RCTs were retrieved. Low-dose colchicine treatment is more suitable for gout patients. Using high-dose colchicine, 40 patients (76.9%) developed diarrhoea, 10 patients (19.2%) developed severe diarrhoea, and nine patients (17.3%) developed vomiting. Using low-dose colchicine, 23.0% of patients developed diarrhoea, and no severe diarrhoea or vomiting occurred. Tuna extracts containing imidazole compounds in anti-hyperuricaemia treatment caused seven adverse events in the low-dose group, 10 in the high-dose group and five in the placebo group. Adverse reactions caused by these two natural drugs should not be ignored. Patients with gout should be given detailed instructions and suggestions for clinical medication. Research on natural drugs for gout is significantly less than that for other skeletal diseases, indicating that there is still enough space for developing new drugs with research value.

Many bone-related diseases require long-term medication. The safety evaluation of natural drugs can provide a detailed medication reference for clinicians, especially in the treatment of ageing-related CSDs. The development and safety evaluation of natural drugs have become a prominent topic in drug research on CSDs. Therefore, because clinical safety evaluation also requires long-term follow-up observation, we have screened out natural medicines that have reported few adverse events, but found that the evaluation time is often very short (<2 years). In order to evaluate their safety more rigorously, a long-term safety evaluation is still needed to verify, and we will continue to pay attention to reports on the safety of these drugs.

## Conclusion

This review summarizes the clinical safety evaluation of multiple natural drugs in the treatment of OP, OA, RA and gout, and lists the clinical trial design, clinical experimental methods and adverse reaction events of patients treated with different natural drugs. Systematic analysis and rational evaluation of natural drugs in clinical adverse event and side effect were carried out to provide evidence for further clinical drug research in CSDs. In addition, clinical safety evaluations of natural drugs in the treatment of CSDs are exiguous. Using traditional literature search channels, it is difficult to obtain more comprehensive clinical trial data of natural drugs for CSDs, because of the following reasons: the complex structure requires more complex production technology, leading to an expensive production cost; the development of natural plants with therapeutic potential to treat CSD are limited by the existing scientific research level and technology; the active components in these natural plants are difficult to determine; The results of clinical trials of some natural drugs are not disclosed. But These results indicate that more clinical trials should be conducted to explore more natural drugs with good pharmacodynamic performance.

## Abbreviations

CSDs, Chronic skeletal disorders; CSE, clinical safety evaluation; RCTs, randomized controlled trials; OP, Osteoporosis; OA, osteoarthritis; RA, Rheumatoid arthritis; GLM, Green Lipped Mussel; BSRE, *Boswellia serrata Roxb* extract; TwHf, *Tripterygium wilfordii Hook.f*; *MTX*, methotrexate; G2013, guluronic acid.
